# Gender Differences in Seeking Health Care and Postintervention Pain Outcomes in Foot and Ankle Orthopedic Patients

**DOI:** 10.1089/whr.2021.0076

**Published:** 2022-05-09

**Authors:** Kyla A. Petrie, Jason N. Chen, Hunter Miears, Jerry Speight Grimes, Mimi Zumwalt

**Affiliations:** ^1^ School of Medicine, Texas Tech University Health Sciences Center, Lubbock, Texas, USA.; ^2^ Department of Orthopedic Surgery, Texas Tech University Health Sciences Center, Lubbock, Texas, USA.

**Keywords:** ankle, foot, gender differences, orthopedics, pain

## Abstract

**Background:**

A significant portion of the adults suffer from foot and ankle pain. The sex differences that exist throughout health care, pain management, and orthopedics may further complicate treatment strategies. The purpose of this study was to determine if there were any differences in women and men in health care seeking behavior and symptom chronicity in a West Texas orthopedic population with foot and ankle conditions.

**Materials and Methods:**

Data from 137 patients were collected in a retrospective chart review. Data were analyzed to determine if there were sex differences in time to primary care provider (PCP) after ankle injury, referral time from PCP to orthopedic surgeon consult, time from orthopedic consult to surgical intervention (if applicable), and chronicity of symptoms.

**Results:**

Women had a significantly higher percentage of chronic injuries than men (30.7% vs. 10.9%), but there were no sex differences in time to PCP from the time of injury, time from PCP visit to orthopedic consult, and time from orthopedic consult to surgical intervention. There were also no differences in those same time frames when compared by chronicity of symptoms (acute injury vs. chronic injury). Finally, we did not find any differences in pain scores between sexes or chronicity of symptoms.

**Conclusions:**

This study suggests that conventional health seeking sex differences may not apply to the foot and ankle patient population in West Texas and surrounding rural areas. Continuing to examine patterns in sex differences may lead to the development of more efficient and tailored treatment approaches and better outcomes.

## Background

Pain is subjective, amorphous, difficult to quantify, and highly individualized to each person who experiences it. There is a tremendous burden on patients to find a way to deal with and manage their pain, especially in a health care system that may not always appreciate the level of pain a patient is truly in.^1,2^ Not only does pain cause problems for individual patients, but musculoskeletal pain is also one of the leading causes of health care and economic expenditure, resulting in missed workdays, disability, and inability to perform recreational activities.^3,4^ It is clear that pain has detrimental effects on both the individual and society as a whole.

There may also be differences in the ways men and women experience pain. For example, women tend to present with pain across different sites of the body, while men tend to localize their pain to one area.^3–7^ Women also report more severe and frequent chronic musculoskeletal pain as well as increased prevalence of postprocedural pain and disability than men.^4,7,8^ This discrepancy holds true for neuropathic pain, with multiple population-based studies showing a nearly twofold increase in the prevalence of chronic neuropathic pain in women compared with men.^7^

One of the most common causes of musculoskeletal pain in any population are forms of arthritis, particularly osteoarthritis, which occurs far more frequently in adults 50 years and older. This age range coincides with the average onset of menopause in women, which through loss of estrogen, causes an increased prevalence of osteoporosis, osteoarthritis, and various other orthopedic complaints.

Multination studies have demonstrated that women on average report greater musculoskeletal and joint pain than men (45% vs. 31%), and present at a later disease state, resulting in an increased pain burden at baseline than their male counterparts.^7,9–11^ Some of this has been attributed to the higher frequency of chronic musculoskeletal pain conditions in women, however, structural and degenerative conditions also seem to occur with higher frequencies in women.^7^ For example, the prevalence of chronic knee pain was found to be higher in females (23.5% vs. 18.1%) and this discrepancy increases as the population ages.^12^ Because of these factors, even when an intervention such as arthroplasty is performed, there is less functional recovery and more pain postsurgically for women compared with men.^10,11,13,14^

Whether this is due to unconscious bias from the initial point of contact with a provider, or an expectation placed on women to portray a façade of strength instead of seeking help for their pain, the fact remains that barriers exist that prevent women from obtaining care for these conditions.^7,15–18^

Foot and ankle complaints are one of the most common orthopedic concerns that affect patients. In middle-aged and elderly populations, up to 20% of people reported that they had foot or ankle pain, with nearly two thirds of those reporting significant pain that limits their daily activities.^19^ The prevalence of pain also tends to be higher in people who are older, and a much larger percentage of women report having foot and ankle pain than their age-matched male counterparts.^19^

Researchers have theorized that the gender differences in foot and ankle pain are related to differences in women's and men's footwear, most notably that women's footwear tends to have greater heel elevation and a more narrow toe box leading to development of various orthopedic disorders.^19^ However, there has been limited focus on studying the differences between men and women regarding seeking help for their foot or ankle pain.

Our Orthopedic Surgery Department receives referrals for musculoskeletal pain from across West Texas and parts of New Mexico, Oklahoma, and Colorado. In this study, our goal was to determine if there were any differences in chronicity of injury based on sex, whether a disparity existed in the time at which women and men were referred to our practices for common complaints of foot and ankle pain, the time it took for an intervention to occur, and if there were any differences in pre- and postintervention pain scores across the groups. To our knowledge, there is no current research on the possible differences in gender on these topics in orthopedics.

We hypothesized that there would be differences in the time it takes for women to first see a primary care provider, get referred to orthopedic specialist care, and time to surgical intervention. We also hypothesized that there would be significant differences in both pre- and postoperative pain scores, with women having higher scores and less pain resolution following intervention. Finally, we hypothesized that more women would have chronic foot and ankle conditions compared with men.

## Materials and Methods

This study is a retrospective review of 137 patients (87 females, 50 males) who were seen in outpatient clinics of our Orthopedic Foot and Ankle Surgeon (J.S.G.). The patients were seen between January 1, 2007 and January 1, 2015. An Institutional Review Board approval was obtained for all aspects of our study. The medical charts were filtered to exclude fractures, cancers, and other associated diagnoses, while focusing solely on foot and ankle pain (chronic in nature or acute injury). Patients' medical record numbers were utilized to look at pertinent findings and were deidentified to protect them in accordance with Health Insurance Portability Accountability Act rules. Final result analysis did not contain any personal patient identifying information.

Both demographic and study-specific data were collected from a retrospective chart review of the orthopedic surgeon's (J.S.G.) charts. Demographic data included year of birth (age), sex, and race. Study-specific data included acute or chronic pain source, lag time from pain onset until being seen by the primary care physician, referral time to get to an orthopedic surgeon, if surgery was done, delayed time until surgical procedure, and pain scales at the preoperative and postoperative visits.

The patient was counted as having a chronic condition if their symptoms had been occurring for greater than or equal to 3 months and an acute condition if their symptoms had been occurring for less than 3 months. Postoperative pain scores were taken from follow-up visits at a minimum of 3 months and a maximum of 6 months, depending on when the patient's visits fell. Referral time was defined as the duration between time at which the patient saw a PCP or Emergency Department health care provider, and the initial visit with an orthopedic surgeon.

Next, we looked at whether the patient underwent surgery, and if so, how long the delay was from the point of initial visit with the orthopedic surgeon to the operation. As a functional outcome, we examined the pain scale ratings at the preoperative and postoperative visits and compared the outcomes between male and female patients. Pain rating scales were classified on a scale of 0 to 10, with 0 being no pain and 10 being defined as the worst pain a patient could imagine.

All data analyses were run using SPSS 26 (IBM, 2020). Two-way analysis of covariance (ANCOVAs) were used to compare the differences between sex, chronicity of the injury, and the time it took to get to a PCP from the time of injury, time from PCP to orthopedic surgeon consult, and time from orthopedic consult to surgical intervention (if there was one). Chi square tests were used to determine if there were any differences between the sexes in chronicity of injuries; if there were differences in sex, chronicity of injury, and racial/ethnic identification; and if there were any gender differences in which patients underwent a surgical intervention.

## Results

Our data set had a total of 137 participants; 87 were women (63.5%). Mean age was 42.6 years (standard deviation [SD] = 14.9). In terms of race/ethnicity, 56.9% (*n* = 78) identified as Caucasian/White, 34.3% (*n* = 47) as Hispanic/Latino, 6.6% (*n* = 9) as African American/Black, 1.5% (*n* = 2) as Asian, and 0.7% (*n* = 1) as Other.

First, we used a chi square analysis to examine the frequency of chronic versus acute injuries by participant race/ethnicity by sex; there was no significant interaction (*χ*^2^ = 0.302, df = 1, *p* = 0.356). There was, however, a significant effect for sex with injury chronicity. Significantly more women than men had chronic injuries (30.7% vs. 10.9%; *χ*^2^ = 4.365, df = 1, *p* = 0.037; Table 1). Given the lack of significance with respect to race/ethnicity, and the significance related to sex, we only examined sex in our subsequent analyses.

**Table 1.** Breakdown of Chronic Injury Versus Acute Injury by Gender

	Women, *n* (%)	Men, *n* (%)	Both
Chronic injury	42 (30.7)	15 (10.9)	57
Acute injury	45 (32.8)	35 (25.5)	80

**FIG. 1.**
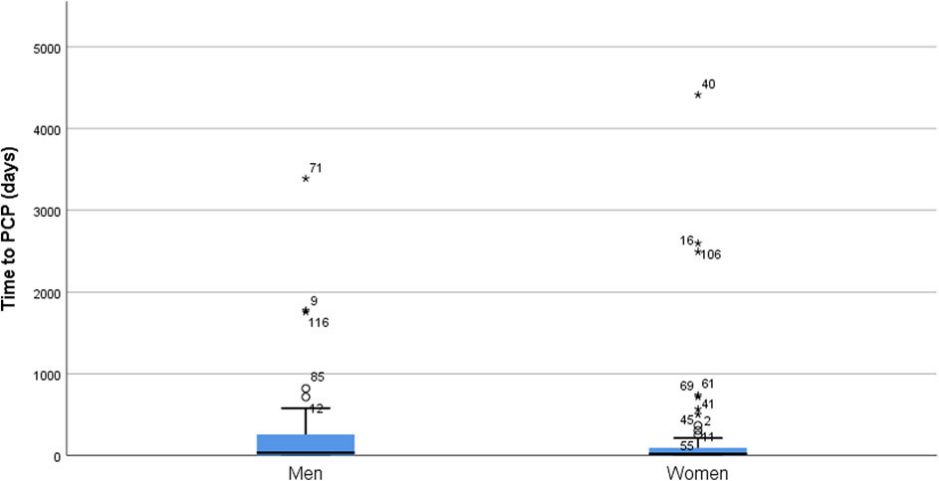
Boxplot of time from onset of injury to being seen by primary care physician by gender.

Using a two-way ANCOVA, we tested the interaction, and main effects, of sex and injury chronicity in relation to time to see a PCP; we entered age as a covariate. Neither the sex by injury chronicity interaction [*F*(1,107) = 0.326, *p* = 0.569, partial *η*^2^ = 0.003] nor the sex main effect [*F*(1,107) = 0.126, *p* = 0.724, partial *η*^2^ = 0.001] were significant. Injury chronicity, however, approached significance [*F*(1,107) = 3.765, *p* = 0.055, partial *η*^2^ = 0.034]; patients with chronic injuries (*M* = 634.76; SD = 1738.92) waited more days to see their PCPs than did those with an acute injury (*M* = 157.04; SD = 517.03l; Table 2 and Fig. 1).

**Table 2.** Average Time by Gender from Symptom Onset to Primary Care Provider (PCP) Visit, PCP Visit to Orthopedic Surgeon Referral Visit, and Orthopedic Surgeon Referral Visit to Intervention (Measured in Days)

	Average time to primary care provider visit from symptom onset (±SD)	Average time from primary care provider visit to orthopedic referral (±SD)	Average time from orthopedic referral to surgery (±SD)
Women (total)	363.14 ± 1380.01	55.06 ± 73.65	119.88 ± 173.45
Acute women	119.21 ± 421.97	55.15 ± 89.89	120.52 ± 138.25
Chronic women	692.37 ± 2026.43	54.93 ± 46.18	125.20 ± 209.79
Men (total)	284.49 ± 632.51	68.67 ± 121.45	109.82 ± 143.14
Acute men	204.65 ± 620.554	59.42 ± 116.94	74.33 ± 73.28
Chronic men	490.75 ± 642.57	92.58 ± 134.73	185.86 ± 222.00

SD, standard deviation.

**FIG. 2.**
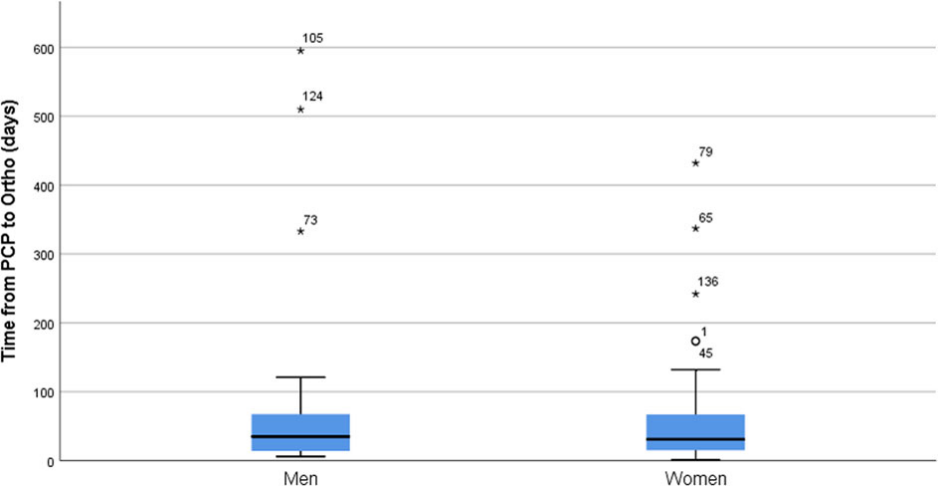
Boxplot of time from visit with primary care physician to being seen by orthopedic surgeon by gender.

We then tested for the main and interactive relationships of sex and injury chronicity to time from PCP to see an orthopedic surgeon; we again controlled for patient age. There were no significant effects: sex by injury chronicity interaction [*F*(1,107) = 0.616, *p* = 0.434, partial *η*^2^ = 0.006], sex main effect [*F*(1,107) = 1.750, *p* = 0.189, partial *η*^2^ = 0.016], and injury chronicity [*F*(1,107) = 0.100, *p* = 0.752, partial *η*^2^ = 0.001; Table 2 and Fig. 2).

**FIG. 3.**
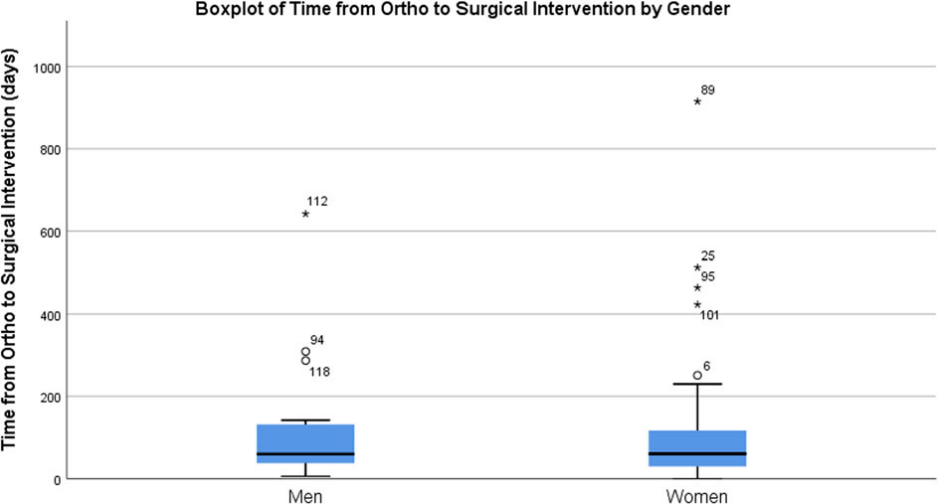
Boxplot of time from initial visit with orthopedic surgeon to surgical intervention (if there was one) by gender.

Finally, we tested the main and interactive relationships of sex and injury chronicity to time from first appointment with orthopedic surgeon to surgery. Again, after controlling for patient age, we found no significant interaction [*F*(1,56) = 1.366, *p* = 0.247, partial *η*^2^ = 0.023], main effects for sex [*F*(1,56) = 0.097, *p* = 0.756, partial *η*^2^ = 0.002], or injury status [*F*(1,56) = 1.406, *p* = 0.240, partial *η*^2^ = 0.024; Fig. 3]. Although the interaction was not significant, likely due to the low power of the test (0.122), there were noticeable differences in the number of days to surgery for a man with an acute injury versus all other groups: acute man (*M* = 74.33; SD = 73.28); chronic man (*M* = 185.86; SD = 222.00); acute woman (*M* = 120.52; SD = 138.25); and chronic woman (*M* = 125.20; SD = 209.79; Table 2).

We did not find any differences between the sexes in pain scores either pre- [*t*(130) = −0.229, *p* = 0.819] or postintervention [*t*(51) = −1.388, *p* = 0.171; Table 3]. We also did not find any differences between sex (*χ*^2^ = 0.125, df = 1, *p* = 0.724) or chronicity of pain (*χ*^2^ = 0.075, df = 1, *p* = 0.784), in terms of which patients received surgery.

**Table 3.** Average Pain Scores by Gender Pre- and Post-Intervention from 0 (No Pain) to 10 (Maximum Pain)

	Average pain preintervention (±SD)	Average pain postintervention (±SD)	Min	Max
Women	6.08 ± 2.55	2.97 ± 3.16	0	10
Men	6.19 ± 2.75	4.25 ± 3.42	0	10

## Discussion

This study reports that there are no significant differences or disparities between men and women in the time from symptom onset to PCP visit, PCP visit to orthopedics referral visit, and from orthopedic visit to surgical intervention (if applicable). Both sexes, regardless of the chronicity of their symptoms, waited similar amounts of time to report their pain to a primary care physician. Although not statistically significant, we want to note that the average amount of days it took a man with a chronic injury to get from orthopedic surgery visit to surgery was 74. For men with chronic injuries, women with acute injuries, and women with chronic injuries, the number was 185, 121, and 125, respectively. We believe that in a further study with a greater number of participants, these numbers may reach significance.

Furthermore, although women were more likely to have a chronic injury, the chronicity of the symptoms did not affect the time from symptom onset to primary care visit, primary care visit to orthopedics referral visit, and from orthopedic visit to surgery. However, chronic injuries, regardless of gender, did approach significance in the time it took for the patient to get to a PCP.

On average, men are twice as likely as women to go a year without visiting a health care provider.^20^ Men being less likely to seek health care may be due to upholding traditional masculine beliefs and social roles.^21–27^ However, in the orthopedic field, men with acute injuries were more likely to report to a primary care physician sooner, while women who had chronic shoulder and knee injuries tended to wait longer to report their pain.^28^

Based on this research, we hypothesized that there would be sex differences when patients seek care in our orthopedic foot and ankle clinic, however, we did not find any such differences. One possible explanation for the deviation of our results from our hypothesis is that musculoskeletal pain in the ankles and feet significantly hinder a patient's ability to walk and proceed with daily activities.^29,30^ Foot and ankle pain are significant risk factors for impaired balance, locomotive disability,^31^ and increased falling risk.^32–34^ Patients may be constantly reminded of their foot and ankle pain, which may lead to earlier physician visits for both sexes, in contrast to preventive health care or conditions that do not impact daily activities.

There were also no significant reported pain differences between men and women, both preintervention and postintervention. Past literature suggests that women report more frequent, longer duration, and more severe pain than men.^7,35–37^ However, these sex differences could be due to variations in pain response between men and women, differences in social norms for pain expression, or differences in biological pain receptors.^38^ Furthermore, the variations in how pain is described, perceived, and quantified may make it difficult to directly compare between patients.

Pain is inherently subjective, and numerical pain scales are influenced by many factors, including personal experiences with pain.^39^ Moreover, although some studies show that women are more likely to report musculoskeletal pain and higher pain intensity than men,^40^ other studies have shown that in the neck, shoulder, back, and knee, there were no significant sex differences in pain prevalence.^38^ Further investigation is warranted to clarify the sex differences in pain severity perception.

A significantly higher proportion of women reported chronic pain compared with men. This is consistent with findings of previous studies and may be due to the higher prevalence of chronic conditions such as osteoarthritis, osteoporosis, and fibromyalgia in women.^41–47^ The significant proportion of women suffering from chronic pain warrants attention because chronic pain has been found to have a negative impact on quality of life.^48^

This study is unique because it focuses on foot and ankle pain, which has a high prevalence of pain but has been given very little attention. Additionally, the majority of the patients in this study were from West Texas and surrounding New Mexico, Oklahoma, and Colorado regions, primarily rural areas. Many patients from more isolated areas lack ready access to health care providers; thus, the time it takes for these patients to seek health care may be different than other studies performed at more urban institutions.^49^ These health care access barriers may hinder proactive behavior, and more focus on rural settings is warranted for examining sex differences in seeking treatment.

There are a few limitations that should be noted with this study. Geographically, we were limited to one institution in West Texas; therefore, generalizability may be limited in larger, more urban settings. Pain intensity scores are commonly used in practices but may have poor sensitivity. For example, the differences in reported postoperative pain among different age groups were better visualized with scales that utilized verbal descriptions of pain qualities rather than numerical pain intensity scales.^50^ Furthermore, because this was a retrospective chart review study, there was no standard phrasing when physicians inquired about patients' pain. We were also limited in our sample size and power, so we hope to investigate this in future research.

## Conclusions

The results of this study help further the current knowledge of sex differences in seeking treatment and reporting pain. We found no significant disparities between men and women in (1) the time it took from symptom onset to seek medical attention from a PCP, (2) the time it took for a referral to an orthopedic surgeon, and (3) the time it took from the orthopedics referral to surgery, if surgery was performed.

While there were no significant differences in the pain severity between men and women, women were found to have chronic pain more often than men. It is important that physicians and public health advocates understand the differences in the health care needs not only of men and women, but also age, race/ethnicity, socioeconomic status, and rural/urban living. Further investigation on sex disparities and the cause of these disparities can help develop sex-specific treatment regimens that may improve outcomes.^51^

## Data Sharing

The datasets used and/or analyzed during the current study are available from the corresponding author on reasonable request.

## Abbreviations Used

ANCOVA: analysis of covariance
PCP: primary care provider
SD: standard deviation
